# Gut microbiome-based machine learning for diagnostic prediction of liver fibrosis and cirrhosis: a systematic review and meta-analysis

**DOI:** 10.1186/s12911-023-02402-1

**Published:** 2023-12-19

**Authors:** Xiaopei Liu, Dan Liu, Cong’e Tan, Wenzhe Feng

**Affiliations:** 1 School of Basic Medicine, Shaanxi University of Chinese Medicine, Xixian Avenue, Xixian New District, Xianyang, 712046 Shaanxi Province China; 2 Xi’an Hospital of Traditional Chinese Medicine, Xi’an, 710016 Shaanxi China; 3 Affiliated Hospital of Shaanxi University of Chinese Medicine, Xianyang, 712046 Shaanxi China

**Keywords:** Gastrointestinal microbiome, Prediction/diagnosis, Liver cirrhosis/fibrosis, Meta-analysis

## Abstract

**Background:**

Invasive detection methods such as liver biopsy are currently the gold standard for diagnosing liver cirrhosis and can be used to determine the degree of liver fibrosis and cirrhosis. In contrast, non-invasive diagnostic methods, such as ultrasonography, elastography, and clinical prediction scores, can prevent patients from invasiveness-related discomfort and risks and are often chosen as alternative or supplementary diagnostic methods for liver fibrosis or cirrhosis. However, these non-invasive methods cannot specify the pathological grading and early diagnosis of the lesions. Recent studies have revealed that gut microbiome-based machine learning can be utilized as a non-invasive diagnostic technique for liver cirrhosis or fibrosis, but there is no evidence-based support. Therefore, this study conducted a systematic review and meta-analysis for the first time to investigate the accuracy of machine learning based on the gut microbiota in the prediction of liver fibrosis and cirrhosis.

**Methods:**

A comprehensive and systematic search of publications published before April 2th, 2023 in PubMed, Cochrane Library, Embase, and Web of Science was conducted for relevant studies on the application of gut microbiome-based metagenomic sequencing modeling technology to the diagnostic prediction of liver cirrhosis or fibrosis. A bivariate mixed-effects model and Stata software 15.0 were adopted for the meta-analysis.

**Results:**

Ten studies were included in the present study, involving 11 prediction trials and 838 participants, 403 of whom were fibrotic and cirrhotic patients. Meta-analysis showed the pooled sensitivity (SEN) = 0.81 [0.75, 0.85], specificity (SEP) = 0.85 [0.77, 0.91], positive likelihood ratio (PLR) = 5.5 [3.6, 8.7], negative likelihood ratio (NLR) = 0.23 [0.18, 0.29], diagnostic odds ratio (DOR) = 24 [14, 41], and area under curve (AUC) = 0.86 [0.83–0.89]. The results demonstrated that machine learning methods had excellent potential to analyze gut microbiome data and could effectively predict liver cirrhosis or fibrosis. Machine learning provides a powerful tool for non-invasive prediction and diagnosis of liver cirrhosis or liver fibrosis, with broad clinical application prospects. However, these results need to be interpreted with caution due to limited clinical data.

**Conclusion:**

Gut microbiome-based machine learning can be utilized as a practical, non-invasive technique for the diagnostic prediction of liver cirrhosis or fibrosis. However, most of the included studies applied the random forest algorithm in modeling, so a diversified prediction system based on microorganisms is needed to improve the non-invasive detection of liver cirrhosis or fibrosis.

**Supplementary Information:**

The online version contains supplementary material available at 10.1186/s12911-023-02402-1.

## Introduction

Liver fibrosis is a pathologic change characterized by an excessive accumulation of liver fibrous tissues resulting from multiple pathogenic factors, including alcohol, medicine, and the hepatitis virus. The unceasing progression of liver fibrosis will cause liver cirrhosis [1], which is a dynamic progress [2]. Unlike liver fibrosis, liver cirrhosis is non-reversible and often causes a poor outcome in cirrhotic patients [3]. Most patients with liver cirrhosis are clinically asymptomatic before the decompensatory stage, so the disease goes undetected and cannot be promptly diagnosed [4]. Therefore, early identification of liver fibrosis is significant. Presently, the gold standard for diagnosing fibrosis is still liver biopsy. However, this invasive detection method may cause such complications as bleeding, biliary peritonitis, and pneumothorax during operation; its histologic assessment may be affected by high sampling-rate errors [5, 6]. Additionally, the liver biopsy must be performed in strict accordance with relevant operation specifications, and its implementation often depends on hospitals’ medical conditions and technological levels. Consequently, this approach cannot be used for large-scale disease screening or assessment in routine care. Although various imaging methods can, to a certain extent, show the grade of liver fibrosis and cirrhosis with relatively reliable accuracy [7], they cannot precisely identify early liver fibrosis [8, 9]. Furthermore, they have some shortages in diagnosis. For instance, magnetic resonance elastography (MRE) requires expensive equipment and specialized knowledge; it sets varying cut-off values of diagnostic data for liver fibrosis caused by different causes. Transient elastography (TE) is less accurate in identifying patients with such complications as ascites and narrow intercostal space [10]. As a result, more accurate non-invasive techniques are urgently needed to address the clinical demand for early detection and severity assessment of liver fibrosis and cirrhosis.

The gut microbiota, a new testing target, has been adopted to diagnose liver fibrosis or cirrhosis in recent years. Gut microbiota analysis, combined with other non-invasive tests, such as Fib-4 and the NAFLD fibrosis score, can detect and stage liver fibrosis or cirrhosis and identify patients at a high risk of progressing to the advanced stage. A previous study found that gut microbiome analysis may be a potential method for diagnosing liver-related diseases with high accuracy [11]. For example, patients with advanced liver fibrosis have fewer clostridium and ruminococci, while cirrhotic patients have more prevotella [12–14]. During the progression of liver fibrosis and cirrhosis, the content of escherichia coli varies before portal hypertension, suggesting that changes in gut microbial are helpful for diagnosing liver fibrosis [15]. In addition, the gut microbiome may play a key role in the disease progression of liver fibrosis or cirrhosis. Muegg et al. [13] found that the α diversity in the gut microbiota significantly declined with the progression of liver cirrhosis, indicating that the changes in the gut microbiota may be closely correlated with the severity of liver cirrhosis. Walker et al. [15] discovered that intrahepatic cholestasis during the progression of liver cirrhosis may be correlated with abundant Gram-negative bacteria, a kind of Salmonella.

Changes in the gut microbiota play a crucial role in developing liver fibrosis or cirrhosis [16]. Recently, the development of machine learning (ML) techniques based on gut microbiome has brought prospects for liver fibrosis and cirrhosis prediction. Common ML algorithms include random forest (RF), logistic regression (LR), decision trees (DT), and support vector machines (SVM). RF, compared to other algorithms, is more advantageous due to high accuracy, a lower risk of over-fitting, automatic selection of important features, and efficient processing of large-scale data sets, which can improve the accuracy and reliability of the diagnostic prediction and reduce the possibility of misdiagnosis [17, 18]. Traditional diagnostic methods for liver disease, such as liver puncture, are usually invasive, whereas ML-based intestinal flora analysis only requires patients’ stool samples and is noninvasive, reducing their discomfort and risks. In addition, it has the potential to identify early signs of liver cirrhosis or fibrosis, facilitate early intervention and treatment, and reduce the risk of disease progression and complications. Meanwhile, it can conduct personalized diagnoses based on each patient’s intestinal microbiome characteristics, helping to develop tailored treatment plans given individual differences [19]. Therefore, this study, for the first time, aims to assess the efficiency of the gut microbiome-based ML model in diagnosing liver cirrhosis and fibrosis, providing new insights into the accurate and non-invasive detection of liver fibrosis and cirrhosis.

## Materials and methods

This meta-analysis was conducted in accordance with the Preferred Reporting Items for Systematic Reviews and Meta-Analyses Statement. The study protocol was designed in the INPLASY - International Platform of Registered Systematic Review and Meta-analysis Protocols (INPLASY202250133).

### Literature search

A systematic search of publications published before April 2th, 2023 in PubMed, Cochrane, Embase, and Web of Science was conducted to collect studies on the application of gut microbiome-based metagenomic sequencing modeling technology to diagnostic prediction of liver cirrhosis or fibrosis. Search terms mainly included Gastrointestinal Microbiome [Mesh], Liver Cirrhosis [Mesh], and Machine Learning [Mesh]. The detailed search strategy is presented in Table S1.

### Literature inclusion and exclusion criteria

The inclusion criteria were as follows:

1. Study type: case-control study, cohort study, nest case-control study, and case-cohort study;
2. Studies that completely constructed a ML model based on intestinal microorganisms for the diagnosis of liver fibrosis or cirrhosis;
3. Studies without external validation were also included in our systematic review;
4. Studies reported in English.

Exclusion criteria were as follows:

1. Study type: meta-analysis, review, guide, and expert opinion;
2. Although a ML model for diagnosing liver fibrosis and cirrhosis was constructed in original studies, its modeling variables did not include intestinal microorganisms;
3. Studies that lack the following outcome indicators for the prediction accuracy of risk models: ROC, c-statistic, c-index, sensitivity, specificity, accuracy, recovery rate, precision rate, confusion matrix, diagnostic four-grid table, F1 score, and calibration curve;
4. Research on single-factor analysis of diagnostic accuracy.

### Literature screening and data extraction

The retrieved studies were imported into the EndNote software. Duplicate publications were marked automatically and manually and then deleted. Titles and abstracts were checked to select potentially eligible studies. Their full texts were downloaded and reviewed to select eligible studies for our systematic review. A basic information spreadsheet was developed before data extraction. The extracted data mainly involved title, first author, publication date, study design, nation, diagnostic criteria, sample size, cohort, model types, external validation, number of types of intestinal microbiota, outcome measures, and information on participants such as mean age and gender. The literature screening and data extraction were completed independently by two researchers, and their results were cross-checked. Any disputes were adjudicated by a third researcher.

### Quality assessment

Two researchers (Xiaopei Liu and Dan Liu) performed the quality assessment for the ten eligible articles using the Quality Assessment of Diagnostic Accuracy Studies-2 (QUADAS-2) tool [20]. The QUADAS-2 tool consists of four domains: index test, patient selection, flow and time, and reference standard. Each domain can be graded as an unclear (yellow), high (red), or low (green) risk bias. Likewise, the applicability is also graded as unclear, high, or low risk in the first three domains [21].

### Statistical methods

In a systematic review based on ML, inconsistent modeling parameters in original studies are a primary source of heterogeneity. Therefore, the present study used the command of “Midas” (StataCorp LLC, College Station, TX) in Stata 15.0 to fit the bivariate mixed-effects model for assessing the sensitivity (SEN), positive likelihood ratio (PLR), specificity (SEP), diagnostic odds ratio (DOR), and negative likelihood ratio (NLR). Furthermore, we calculated estimates with 95% confidence intervals (95% CIs), drew the summary receiver operating characteristic curves (SROC), and calculated the area under curve (AUC) with 95% CI. Deek’s funnel plot was employed to detect publication bias, and Q statistic and I^2^ statistic were used for the heterogeneity test. An *I*^*2*^ > 50% indicated significant heterogeneity. A value of *p* less than 0.05 was considered statistically significant. A value of *p* more than or equal to 0.05 indicated no statistical difference.

## Results

### Literature retrieval results

**Fig. 1:**
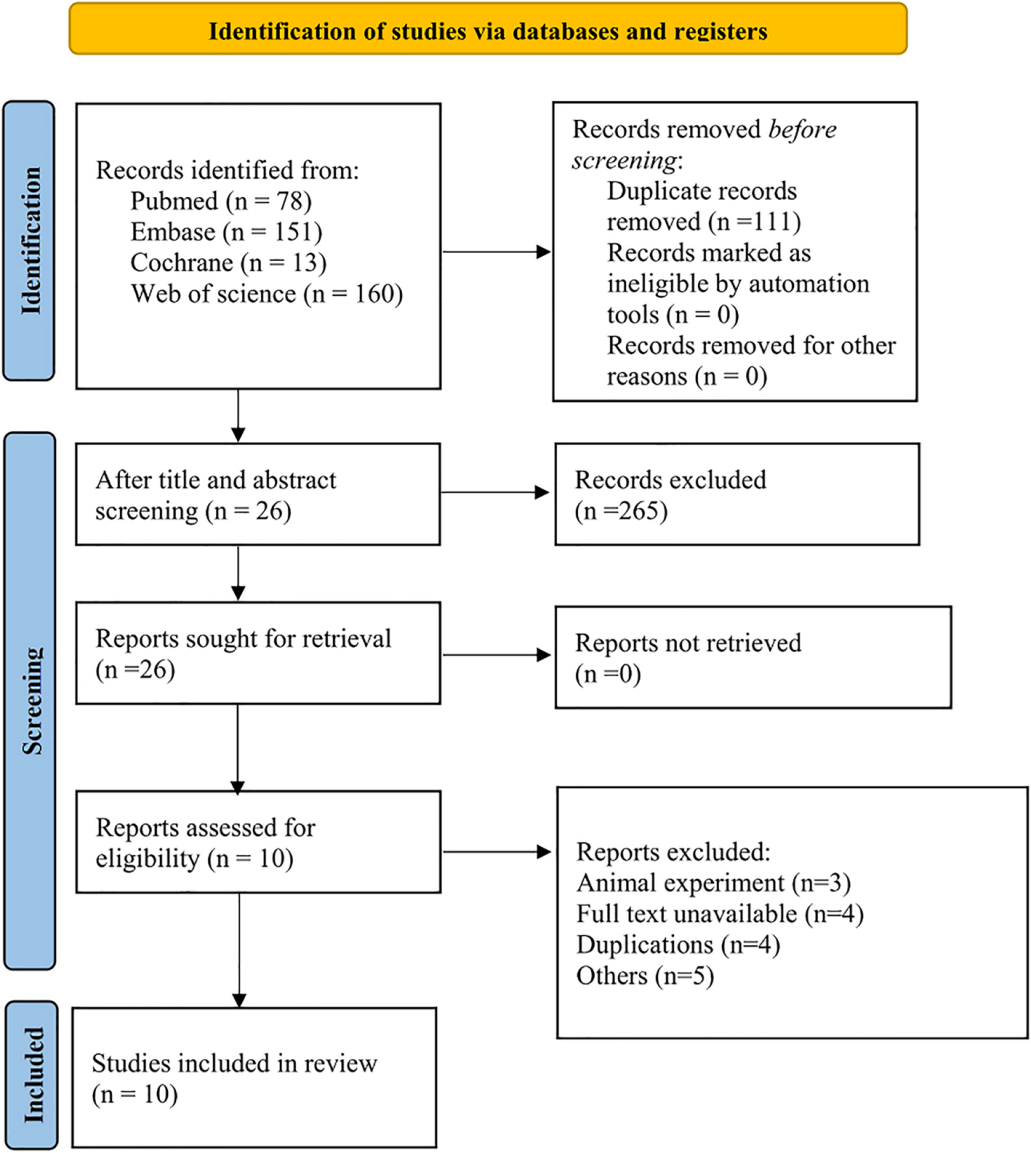
Flowchart of the literature selection

Initially, 402 articles were identified in database searches, and duplicates were removed using the EndNote software. After the titles and abstracts screening, there were 26 articles left. Based on a full-text review, ten studies comprising 11 prediction trials were finally included [22–31]. The literature selection process is shown in Fig. 1.

### Basic characteristics and quality assessment of included literature

**Fig. 2:**
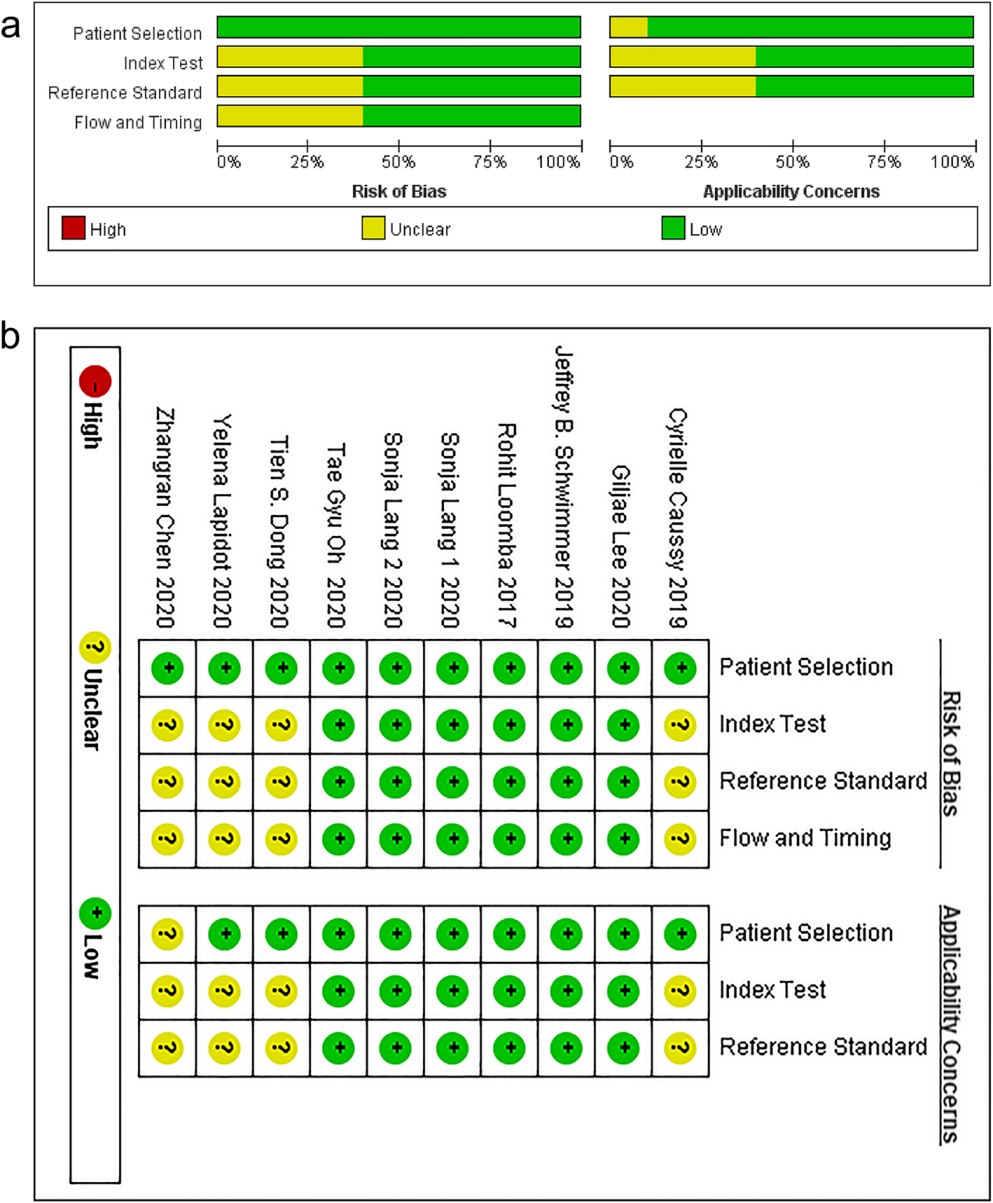
Quality assessment of the included studies. Risk bias and applicability concerns graph (**a**). Risk bias and applicability concerns summary (**b**). The red, yellow, and green colors indicate high, unclear, and low risk, respectively

This meta-analysis included ten articles involving 11 trials and 838 patients. The main ML algorithm was RF. Five trials conducted external validations, and all the included trials were considered high quality. The basic characteristics of included trials are presented in Tables 1 and 2. The quality assessment results are shown in Fig. 2.

**Table 1:** Baseline characteristics

Author	year	country	DET	DIT	NO.TC	NO.TTC	DM	ES	AM
Caussy	2019	USA	cohort study	cirrhosis	26	72	16S rRNA	27 Genus	MRI
Dong	2020	USA	control trial	fibrosis	50	75	16S rRNA	26 Genus	UE
Chen	2020	CHN	control trial	cirrhosis	25	97	16S rRNA	32 Genus	MRI + CT
Gyu Oh	2020	USA	cohort study	cirrhosis	27	81	Shotgun metagenomic	37 Genus	Liver Biopsy
Loomba	2017	USA	cohort study	fibrosis	14	86	Whole genome shotgun	37 Genus	Liver Biopsy
Lang 1A	2020	USA	cohort study	cirrhosis	44	73	Shotgun metagenomic	420 Species	Liver Biopsy
Lang 1B	2020	USA	cohort study	fibrosis	29	73	Shotgun metagenomic	420 Species	Liver Biopsy
Lang 2	2020	DEU	cohort study	fibrosis	13	96	16S rRNA	37 Genus	Liver Biopsy
Lapidot	2020	ISR	control trial	cirrhosis	68	95	16S rRNA	/	Based on histological and/or clinical findings
Lee	2020	Kr	control trial	fibrosis	64	117	16S rRNA	2 Family	Liver Biopsy
Schwimmer	2019	USA	control trial	cirrhosis	87	124	16S rRNA and Metagenomics	3 Phylum	Liver Biopsy

*DET* Designing Types, *DIT* Diagnostic Target, *NO. TC* Number of cirrhosis/fibrosis cases in training cohort, *NO. TTC* The total number of cases in the training cohort, *DM* Detection method, *ES* Enterobacteriaceae species, *AM* Assessment Method, *MRI* Magnetic Resonance Imaging, *UE* Ultrasound Elastography, *CT* Computed Tomography

**Table 2:** Baseline characteristics

Author	Year	Sex	Age (year)	MT	ODM	ROC	TP	FP	FN	TN
Caussy	2019	9/29	65.1 ± 9.8	Random forest	/	/	22	7	4	39
Dong	2020	19/0	66.2 ± 6.8	Random forest	10-fold cross-validation	0.82	36	9	14	16
Chen	2020	20/5	51.24 ± 6.91	Random forest	10-fold cross-validation	/	20	5	5	46
Gyu Oh	2020	5/22	64.74 ± 9.80	Random forest	10-fold cross-validation	Italy 0.89China 0.88	23	8	4	19
Loomba	2017	2/13	63.4 ± 3	Random forest/ Support Vector Machines	Separation of training set and test set	/	13	4	1	68
Lang 1A	2020	20/24	58.9 (20.2–79.6)	Logistic model	Leave-One-Out Cross-Validation	/	8	9	1	55
Lang 1B	2020	16/13	51.9 (28.8–74.2)	Logistic model	Leave-One-Out Cross-Validation	0.71	21	5	8	39
Lang 2	2020	8/5	64.0 ± 7.0	Random forest	Separation of training set and test set	/	12	17	1	66
Lapidot	2020	46/22	65.9	Random forest	20-fold cross-validation	/	62	10	6	17
Lee	2020	27/37	58.7 ± 10.7	/	/	0.721	48	6	16	101
Schwimmer	2019	62/65	12 ± 10.7	Classification Regression Tree/Decision Tree	10-fold cross-validation	/	65	1	22	36

*Sex* Male/Female, *MT* Model type, *ROC* External Verification (Roc), *TN* True negative, *TP* True positive, *FN* False negative, *FP* False positive, *ODM* Overfitting detection method

### Results of meta-analysis

#### Overall statistics of liver fibrosis and cirrhosis

**Fig. 3:**
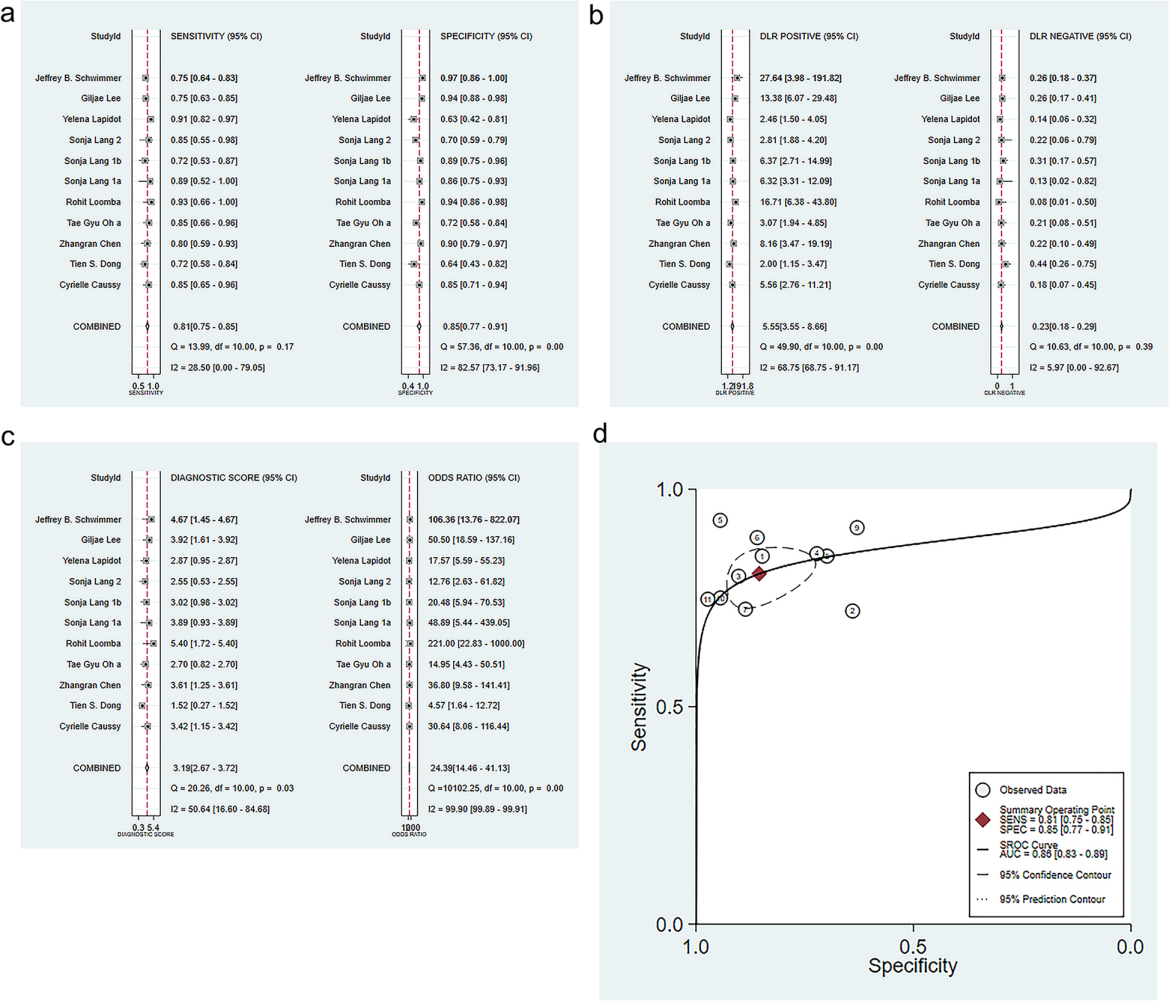
Forest plot of SEN and SPE for the diagnosis of liver fibrosis and cirrhosis using gut microbiome-based ML (**a**); Forest plot of likelihood ratios for the diagnosis of liver fibrosis and cirrhosis gut microbiome-based ML (**b**); Forest plot of DOR for the diagnosis of liver fibrosis and cirrhosis using gut microbiome-based ML (**c**); Forest plot of SROC for the diagnosis of liver fibrosis and cirrhosis using gut microbiome-based ML (**d**). In figure (**d**), the full line represents the SROC curve; numerical circles represent the included prediction trials; the red rhombus indicates the point estimate of sensitivity/specificity; and the dotted line indicates the 95% confidence intervals (95% CI: 0.83–0.89)

A bivariate random-effects model was employed to pool statistics. The results were: SEN = 0.81 (95% CI: 0.75–0.85), SPE = 0.85 (95% CI: 0.77–0.91), PLR = 5.55 (95% CI: 3.55–8.66), NLR = 0.23 (95% CI: 0.18–0.29) (Fig. 3a-c), DOR = 24.39 (95% CI:14.46–41.13) and SROC = 0.86 (95% CI: 0.83–0.89). The results suggested that the overall diagnostic accuracy was high (Fig. 3d).

#### Summary statistics for liver fibrosis and cirrhosis as well as liver biopsy diagnoses

A bivariate random effects model was adopted to pool the statistics. The results were as follows: (1) Liver fibrosis: SEN = 0.77 (95% CI: 0.69–0.82), SPE = 0.84 (95% CI: 0.71–0.92), and SROC = 0.80 (95% CI: 0.76–0.83); (2) Liver cirrhosis: SEN = 0.84 (95% CI: 0.77–0.891), SPE = 0.85 (95% CI: 0.74–0.92), and SROC = 0.91 (95% CI: 0.88–0.93); (3) Liver biopsy: SEN = 0.82(95% CI: 0.76–0.88), SPE = 0.87(95% CI: 0.76–0.93), and SROC = 0.89(95% CI: 0.86–0.92). (Figure S1a-d and Figure S2a, b). The results indicated that gut microbiome-based ML was more efficient in diagnosing liver cirrhosis than liver fibrosis. Excluding studies with non-liver biopsy diagnosis had no significant impact on the original results.

### Heterogeneity test

**Fig. 4:**
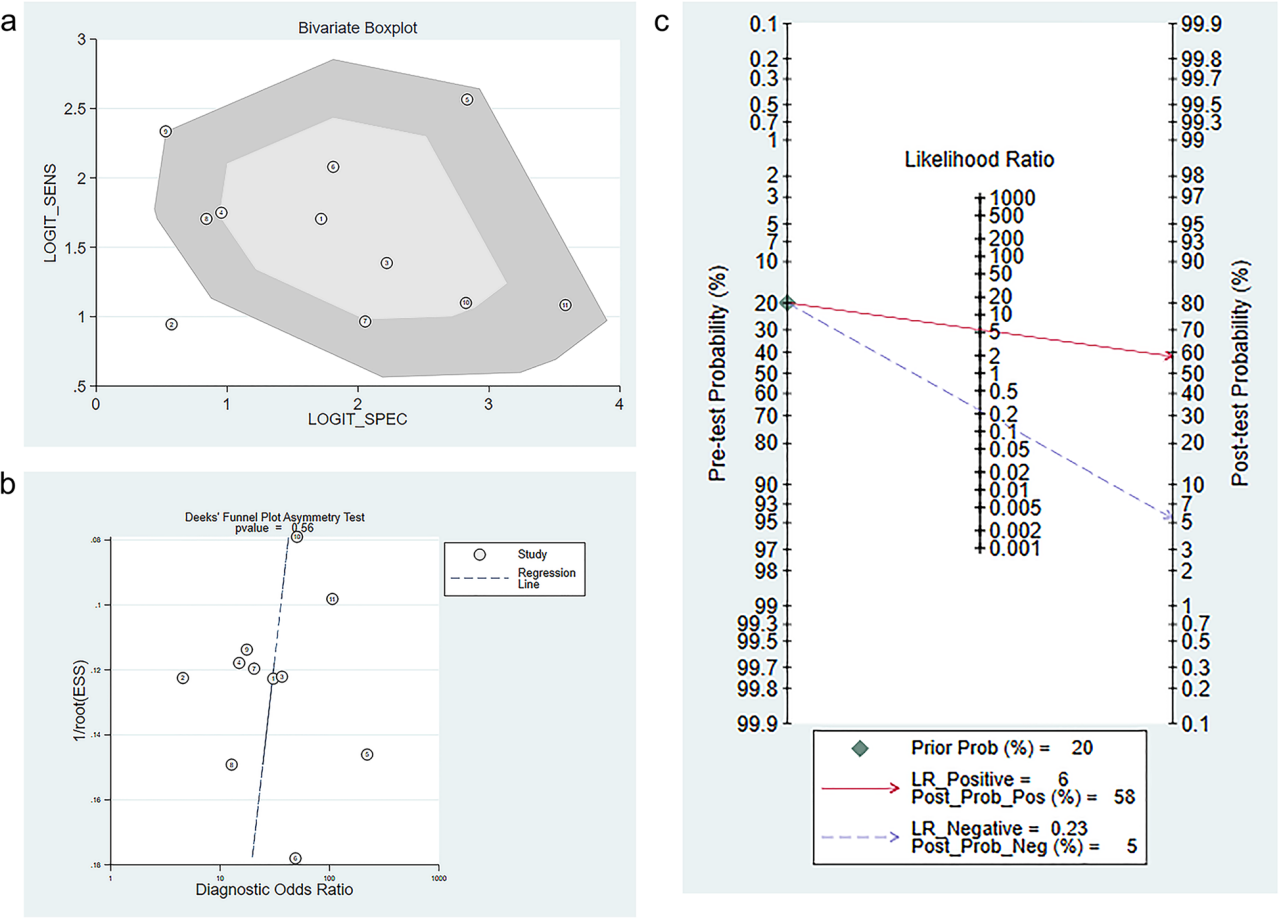
Bivariate box plot for the diagnosis of liver fibrosis and cirrhosis using gut microbiome-based ML (**a**); Deek’s funnel plot for the diagnosis of liver fibrosis and cirrhosis gut microbiome-based ML (**b**); Fagan diagram for the diagnosis of liver fibrosis and cirrhosis using gut microbiome-based ML (**c**). In figure (**b**), numerical circles represent the included prediction trials; the dotted line represents the regression line. In figure (**c**), the green rhombus represents the prior probability; the full red line represents the probability of the diagnosis of liver fibrosis and cirrhosis; the gray dotted line represents the probability of having liver fibrosis or cirrhosis

The heterogeneity was considered significant [I^2^ = 91%, (95%CI: 83–100)]. As shown in Fig. 4a, two of the 11 included trials were located outside the box diagram, indicating that these two trials might be the primary sources of heterogeneity. To further examine whether different diagnostic criteria have an impact on heterogeneity, heterogeneity test was performed again after excluding non-liver biopsy studies. The results showed I^2^ = 93, 95% CI = [86–99], as shown in Supplementary Fig. S4.

### Publication bias

According to Deek’s funnel plot (Fig. 4b), no significant publication bias was observed (*p* = 0.56). This suggested that our meta-analysis results were reliable.

### Verification for diagnostic efficiency

The posterior probability was calculated according to the Fagan diagram (Fig. 4c). If the immediate probability was 20%, the probability of gut microbiome-based ML for diagnosing liver fibrosis and cirrhosis was 58%. If a negative result was reached, the probability of existing liver fibrosis or cirrhosis was 5%. The results indicated that gut microbiome-based ML could be remarkably accurate in diagnosing liver fibrosis or cirrhosis.

## Discussion

Gut microbiota, also known as intestinal flora or intestinal microecosystem, refers to the microbial community in the intestines, including bacteria, fungi, viruses, and other microorganisms [32]. Researchers have become increasingly interested in the association between gut microorganisms and liver diseases. That is because the liver has the closest correlation with the gut and is easily exposed to many bacterial components and metabolites. Gut microbes interact and restrict each other to maintain the homeostatic balance of intestinal flora. When the homeostasis becomes unbalanced, the disordered flora can interact with the host immune system through the portal vein and bile secretion system, resulting in the occurrence and progression of hepatitis, fibrosis, and cirrhosis [32, 33]. The changes in the intestinal flora may provide novel insights into the exploration of new medical interventions. Several studies have proved that gut microbiota can be a diagnostic and therapeutic target for liver diseases [34, 35]. These findings suggest that gut microbiome-based ML models have enormous potential for the diagnosis of liver fibrosis and cirrhosis. Compared to non-invasive diagnostic methods such as ultrasonography, elastography and clinical predictive scoring, gut microbiome-based diagnostic techniques have some potential advantages in diagnosing liver fibrosis and cirrhosis. Especially, such techniques can provide early biomarkers to facilitate early diagnosis and intervention, whereas traditional methods may be not able to detect diseases until they progress to a later stage or symptoms appear [19].

## Main findings

Ten studies were included in this systematic review, and one of them involved two prediction trials. Therefore, the present study involved 11 prediction trials and 838 patients, including 403 patients with liver cirrhosis or fibrosis and 435 patients with other diseases. The present study showed the gut microbiome-based ML method has high sensitivity, specificity, and accuracy in predicting liver fibrosis or cirrhosis (sensitivity: 0.81, specificity: 0.85, AUC: 0.86). In clinical practice, a high AUC (Area Under the Curve) value indicates better predictive performance of the model. However, it is essential to consider sensitivity and specificity along with it. An AUC of 0.86, a sensitivity of 0.81, and a specificity of 0.85 indicate excellent predictive capabilities, leading to improved clinical diagnostic performance. This suggests that machine learning methods based on gut microbiota show strong predictive performance for liver cirrhosis and fibrosis. It implies that a machine learning-based gut microbiome analysis, as a non-invasive diagnostic approach for liver cirrhosis and fibrosis, holds significant clinical applicability and can offer better decision-making support for healthcare professionals.

According to the verification results, in the case of liver fibrosis or cirrhosis diagnosis based on gut microbes, the probability of liver fibrosis or cirrhosis is 85%; otherwise, a negative result indicates a 19% chance of liver fibrosis or cirrhosis. The results reveal that the gut microbiome-based ML method is highly precise for diagnosing liver fibrosis or cirrhosis. The subgroup analysis by liver cirrhosis and fibrosis showed that the SEN, SPE, and SROC for liver fibrosis were 0.77, 0.87, and 0.80, respectively; the SEN, SPE, and SROC for liver cirrhosis were 0.84, 0.85, and 0.90, respectively. These results reveal that the gut microbiome-based ML method is more accurate in diagnosing liver cirrhosis than liver fibrosis. This finding is also confirmed by the research of Tien S. Dong et al. [23]. They compared patients with advanced liver fibrosis with those with mild liver fibrosis and found that patients with advanced fibrosis had obvious microbiota characteristics, and this result was not affected by the cause of liver disease. After adjusting for other covariates, this conclusion is established. The SEN, SPE, and SROC for liver biopsy were 0.82, 0.87, and 0.89, respectively. However, there was no significant difference between the diagnostic performance of liver biopsy and overall diagnostic performance, suggesting that excluding studies with non-liver biopsy diagnostic criteria had no significant impact on the diagnostic performance of gut microbiome-based machine learning methods. These results are visualized in a histogram (Figure S3), which can intuitively reflect their differences.

In contrast, non-invasive diagnostic methods, such as ultrasonography, elastography, and clinical predictive scoring systems, protect patients from invasiveness-related discomfort and risks and are often used as alternative or complementary tools for diagnosing liver fibrosis or cirrhosis. Elastography has become a preferred non-invasive imaging technique for the clinical assessment of liver fibrosis and cirrhosis. Liver stiffness measurement (LSM) provided by elastography is an alternative quantitative biomarker for fibrosis and cirrhosis burden in chronic liver disease. Elastography can be performed with ultrasound or MRI. TE (FibroScan, Echosens) is an ultrasound-based imaging technology most commonly used to assess fibrosis in patients in clinical practice. The AUROC value of TE for cirrhosis diagnosis (0.95–0.97) was higher than that for significant fibrosis (0.80) [36], which is consistent with the results of our subgroup analysis (Supplementary Fig. S1). Magnetic resonance (MR) elastography has been shown to accurately diagnose liver fibrosis and cirrhosis. MR elastography is similarly accurate in diagnosing significant fibrosis and cirrhosis. A meta-analysis of nearly 700 patients found that the mean AUCs of MR Elastography were 0.84, 0.88, 0.93, and 0.92 for stages 1–3 fibrosis as well as cirrhosis, respectively [37]. In summary, in addition to gut microbiome-based ML methods, other non-invasive diagnostic techniques are also accurate in the diagnosis of liver fibrosis and cirrhosis.

Although microflora is largely influenced by genetics [38], dynamic environment [39], seasons [40], diet [41], and smoking [42], the gut microbes are specific in predicting liver fibrosis and cirrhosis. Boursier et al. [43] discovered that patients with non-alcoholic steatohepatitis (NASH) or fibrosis had considerably higher levels of ruminococcus. This result was proved by Bajaj JS [44]. Oh et al. [25] found that a core set of gut microbiome species can detect cirrhosis in patients from geographically separated regions, free from the effects of disease etiology, environmental factors, and host genetics on the gut microbiome. Five of the included studies [23, 25, 26, 28, 30] contain six cohorts, and all of them conducted external validations of diagnostic efficiency. The AUCs of the six cohorts are 0.82, 0.89, 0.88, 0.81, 0.71, and 0.721, respectively. These cohorts comprise patients of different ethnic groups, environments, and etiologies. This indicates that gut microbiota signatures are robust in detecting liver fibrosis and cirrhosis in patients of different settings and etiologies. The gut microbiome-based ML method is universal and stable in diagnosing liver fibrosis and cirrhosis. Furthermore, a Deek’s funnel plot was drawn to evaluate the publication bias. The nearly symmetrical plot indicates no publication bias (*P* = 0.56 > 0.10), suggesting that our results are reliable.

### Implications for clinicians, policymakers and other researchers

Although there are few studies on the application of gut microbiota in diagnosing liver cirrhosis or liver fibrosis, we still found that the gut microbiota has a strong association with the pathological grading of liver cirrhosis or liver fibrosis, indicating its great performance in diagnosing liver fibrosis and early liver cirrhosis. In addition to offering references for non-invasive diagnoses of liver cirrhosis and fibrosis, it can be combined with other non-invasive serum/plasma or imaging tests to stage liver fibrosis and cirrhosis clearly or to evaluate the effects of treatment dynamically. However, the clinical application of this technique requires clinicians equipped with extensive experience. Therefore, it is advised to strengthen operator training, improve the quantity and quality of original studies, including randomized controlled trials and multi-centered studies with large sample sizes and diversified ML algorithms, optimize predictive efficiency, and reduce the bias due to modeling with a single algorithm. In this way, research evidence would be more scientific, accurate, and of high quality, providing meaningful guidelines for clinical decision-making.

## Research limitations

The application of the gut microbiome-based ML method in diagnosing liver fibrosis or cirrhosis is recommendable, but the present study still has some limitations.

1. The difference between operators affected the predictive results. Removing the examination results by inexperienced operators could avoid artificial influences caused by the batch processing effect in any single data set, which can improve accuracy and diagnostic performance. In an analysis of fecal NAFLD metagenomes based on three publicly available studies, Wang et al. [16] reported that the differences in metagenomic methods and study designs (e.g., sample collection and preservation, sequencing platforms, and DNA extraction methods) could impact the constitution of downstream sequential data. The three studies (Illumina HiSeq) used the same sequencing platforms, but their DNA extraction methods differed. As all three studies explained that their samples were extracted by fast freezing to − 80 °C, there are still technical variances due to human factors.
2. Differences in sequencing methods had an impact on the results. High-throughput sequencing of the culture-independent 16S rRNA gene is the primary research method. However, with the sequencing costs increasing and the data analysis methods maturing, metagenomic shotgun sequencing is expected to become an essential means of studying the gut microbiome due to its advantages of processing larger-scale and comprehensive information. Most included studies adopted the 16S ribosomal RNA sequencing method; one used shotgun metagenomic sequencing [24]; and two used whole-metagenome shotgun sequencing [23, 25]. Different sequencing methods also caused heterogeneity in research results, which was reported in the study by Bajaj JS et al. [44].
3. Undiversified models may also affect the research results. The main ML algorithm used in the included studies was RF. As a dynamic and complex ecosystem in the human body, the gut microbiota is affected by the interactions within microbial colonies as well as between microbial colonies and hosts. It is difficult for undiversified models to present such complicated interactions. Therefore, the multi-omics integration strategy will be a promising approach to understanding the gut microbiota composition and physiological functions.
4. The research results were also affected by sample size. The application of gut microbiota in diagnosing liver cirrhosis or liver fibrosis is still in the primary stage. At present, only few studies have been published on the gut microbiome-based ML method predicting liver fibrosis and cirrhosis. Thereby, there was a small sample size in the present study, and it is impossible to perform subgroup analysis according to microbial species. More studies with a larger sample size are needed, and we will supplement and update the analysis in a timely manner.
5. The impact of heterogeneity on research results

The heterogeneity test indicates the existence of heterogeneity (*I*^*2*^ = 63,95% CI = [16–100]). As shown in the bivariate box plot, 2 of the 10 included studies were located outside the box plot, suggesting that these two studies may be the main source of heterogeneity. After carefully reading these two studies, we found that their sample size and distribution were different, which may affect the performance and applicability of each model. Furthermore, the feature selection method, study type, and study population may all cause heterogeneity. In some countries, liver fibrosis or cirrhosis is not diagnosed by liver biopsy, for example, the three included studies by Caussy, Dong, and Chen. To delve into whether they have an impact on heterogeneity, we used the numerical method and a bivariate box plot to test heterogeneity after excluding the three studies that did not use liver biopsy to diagnose liver fibrosis or cirrhosis. The results showed I^2^ = 93, 95% CI = [86–99], suggesting that these three studies may not be the source of heterogeneity, and differences in diagnostic criteria have no significant impact on the study results.

(6) Impact of external validation on results

In bioinformatics studies, external validation is an important step to ensure the reliability and applicability of research results, making such studies more scientific and practical. In our analysis, most included studies only used K- fold cross validation or random sampling methods to generate validation sets, but did not evaluate the generalization ability of their models or results in new data sets. This may limit the interpretation of the results. Therefore, more follow-up studies are needed to verify our findings.

## Conclusion

The gut microbiome-based ML method can effectively predict liver cirrhosis and fibrosis. It is an optimal approach for non-invasive diagnosis of liver cirrhosis and fibrosis with a high clinical application value. Nevertheless, the present study merely included few studies and undiversified models. Therefore, developing a diversified prediction system based on microorganisms is necessary to enrich the efficient non-invasive detection methods for liver cirrhosis or fibrosis.

The introduction of diverse gut microbiome in machine learning will enhance model performance, robustness, and applicability, reduce potential bias in models, improve the interpretability of models, provide more information, and help clinicians make more comprehensive decisions.

### Outlook

In summary, ML methods based on intestinal microorganisms have broad potential and prospects in the diagnosis of cirrhosis and liver fibrosis due to noninvasiveness, early diagnosis, personalized diagnosis and treatment, data integration, reduced medical costs, and interdisciplinary cooperation. However, challenges such as data quality, model interpretation, and clinical validation still need to be addressed in the future. Overall, with constant advances in technology and deepening research, ML will continue to play a key role in improving the diagnosis and treatment of liver diseases.

### Supplementary Information

**Additional file 1.**

**Additional file 2.**

## Data Availability

Data sharing is not applicable to this article as no datasets were generated or analysed during the current study.
