# Supplementary material for: Gut microbiome-based machine learning for diagnostic prediction of liver fibrosis and cirrhosis: a systematic review and meta-analysis
Source: BMC Med Inform Decis Mak. 2023 Dec 19;23:294. doi: 10.1186/s12911-023-02402-1 (PMC10731850; doi:10.1186/s12911-023-02402-1)
Supplement: Supplementary file 1 — Additional file 1. [file 12911_2023_2402_MOESM1_ESM.docx]

# Supplementary Materials


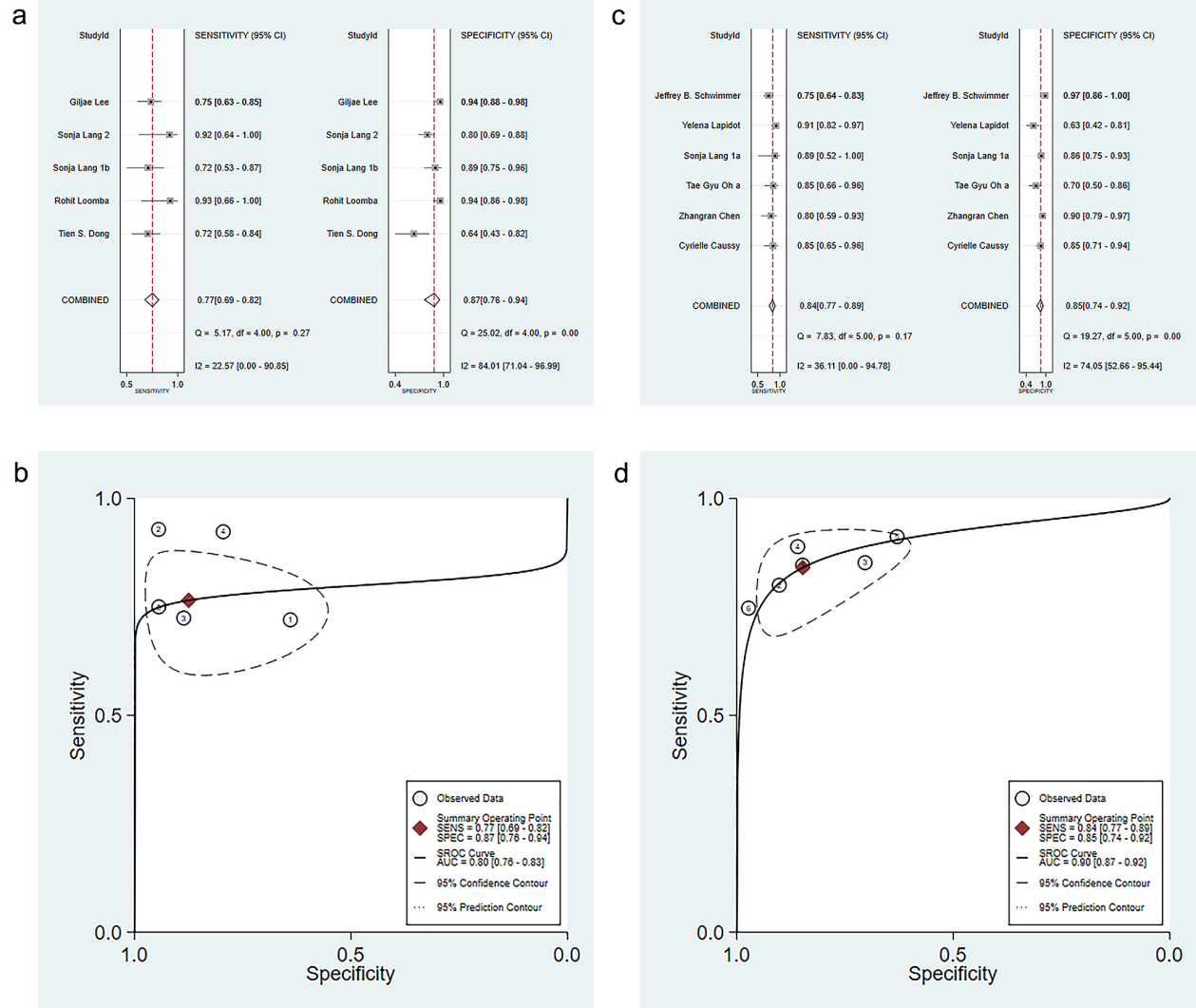


**Supplementary Figure S1** Forest plot of SEN and SPE for the diagnosis of liver fibrosis using gut microbiome-based ML (a); Forest plot of SROC for the diagnosis of liver fibrosis using gut microbiome-based ML (b); Forest plot of SEN and SPE for the diagnosis of liver cirrhosis using gut microbiome-based ML (c); Forest plot of SROC for the diagnosis of liver cirrhosis using gut microbiome-based ML(d). In figures (b) and (d), the full line represents the SROC curve; numerical circles represent the included prediction trials; the red rhombus represents the point estimate of sensitivity/specificity; and the dotted line indicates the 95% confidence intervals (95% CI: 0.74-0.92 and 0.88-0.93, respectively).


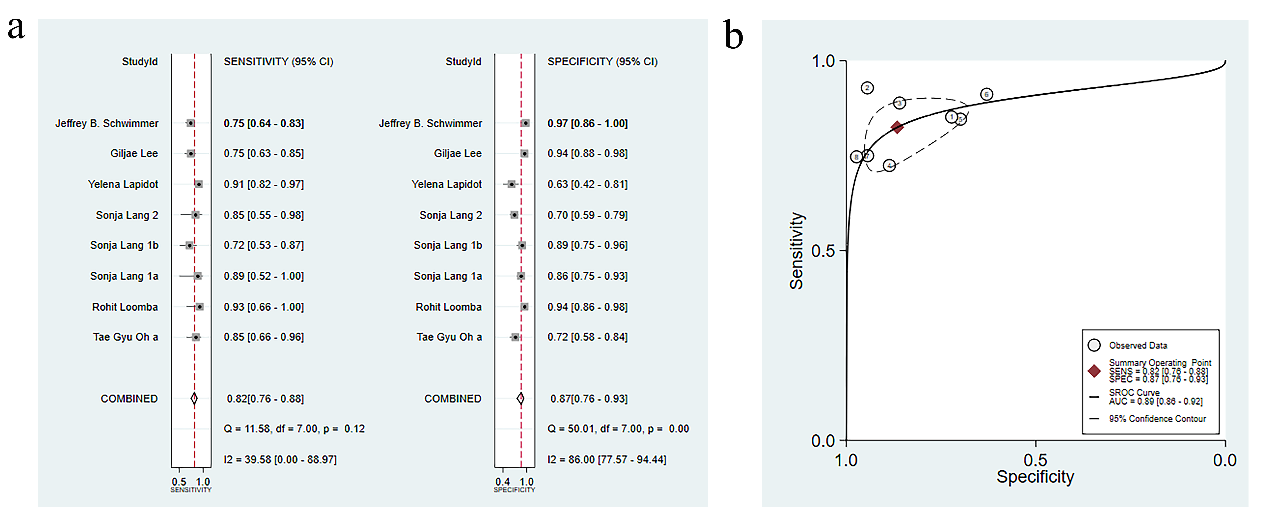


**Supplementary Figure S2** Forest plot of SEN and SPE for ML diagnosis of liver fibrosis and cirrhosis using gut microbiome with liver biopsy diagnostic criteria (a); SROC forest plot for diagnosis of liver fibrosis and cirrhosis using gut microbiome-based ML using liver biopsy diagnostic criteria (b). In figures (b), the full line represents the SROC curve; numerical circles represent the included prediction trials; the red rhombus represents the point estimate of sensitivity/specificity; and the dotted line indicates the 95% confidence intervals (95% CI:0.86-0.92).


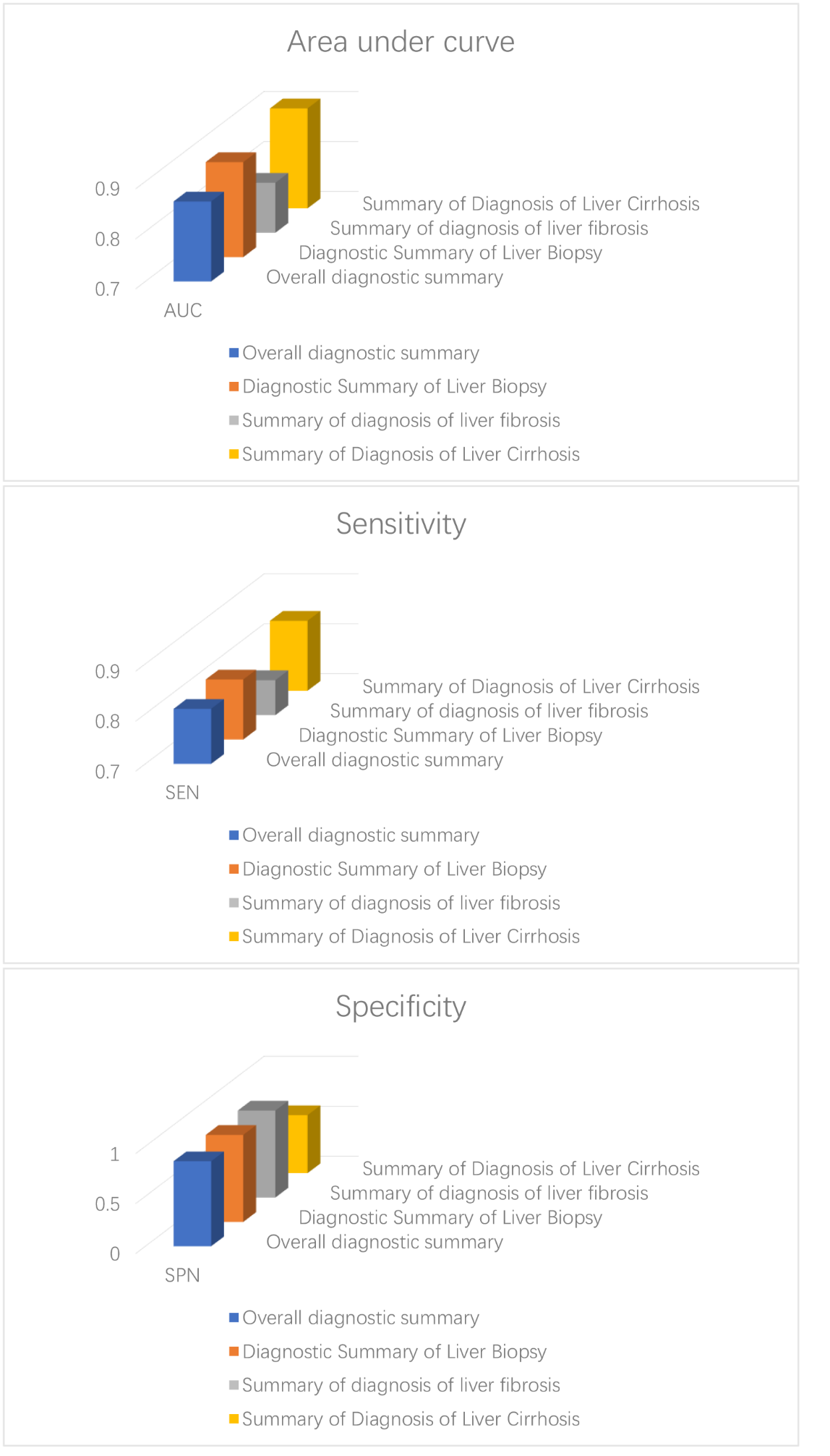


**Supplementary Figure S3 Results comparison bar chart**


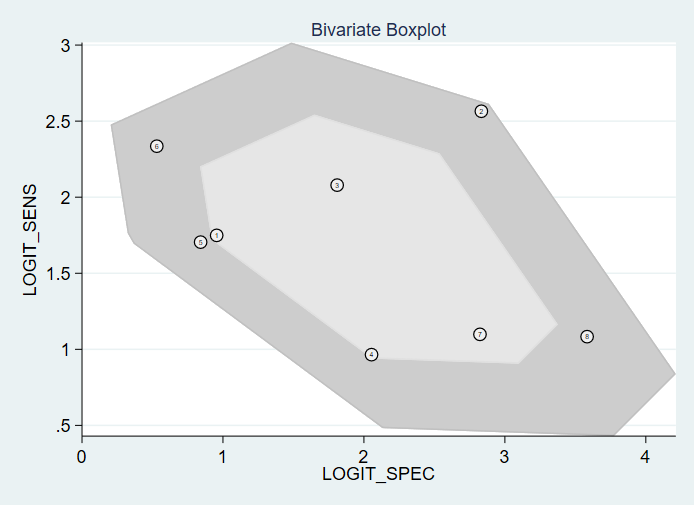


**Supplementary Figure S4** Bivariate box plot of liver biopsy heterogeneity test based on diagnostic criteria.

**Supplementary TableS1** Literature search strategy

**1.Pubmed**

| Search number | Query | Results |
| --- | --- | --- |
| #1 | "Gastrointestinal Microbiome"[Mesh] | 27,757 |
| #2 | ((((((((((((((((((((((((((((((((((((((Gastrointestinal Microbiome[Title/Abstract]) OR (Gastrointestinal Microbiomes[Title/Abstract])) OR (Microbiome, Gastrointestinal[Title/Abstract])) OR (Gut Microbiome[Title/Abstract])) OR (Gut Microbiomes[Title/Abstract])) OR (Microbiome, Gut[Title/Abstract])) OR (Gut Microflora[Title/Abstract])) OR (Microflora, Gut[Title/Abstract])) OR (Gut Microbiota[Title/Abstract])) OR (Gut Microbiotas[Title/Abstract])) OR (Microbiota, Gut[Title/Abstract])) OR (Gastrointestinal Flora[Title/Abstract])) OR (Flora, Gastrointestinal[Title/Abstract])) OR (Gut Flora[Title/Abstract])) OR (Flora, Gut[Title/Abstract])) OR (Gastrointestinal Microbiota[Title/Abstract])) OR (Gastrointestinal Microbiotas[Title/Abstract])) OR (Microbiota, Gastrointestinal[Title/Abstract])) OR (Gastrointestinal Microbial Community[Title/Abstract])) OR (Gastrointestinal Microbial Communities[Title/Abstract])) OR (Microbial Community, Gastrointestinal[Title/Abstract])) OR (Gastrointestinal Microflora[Title/Abstract])) OR (Microflora, Gastrointestinal[Title/Abstract])) OR (Gastric Microbiome[Title/Abstract])) OR (Gastric Microbiomes[Title/Abstract])) OR (Microbiome, Gastric[Title/Abstract])) OR (Intestinal Microbiome[Title/Abstract])) OR (Intestinal Microbiomes[Title/Abstract])) OR (Microbiome, Intestinal[Title/Abstract])) OR (Intestinal Microbiota[Title/Abstract])) OR (Intestinal Microbiotas[Title/Abstract])) OR (Microbiota, Intestinal[Title/Abstract])) OR (Intestinal Microflora[Title/Abstract])) OR (Microflora, Intestinal[Title/Abstract])) OR (Intestinal Flora[Title/Abstract])) OR (Flora, Intestinal[Title/Abstract])) OR (Enteric Bacteria[Title/Abstract])) OR (Bacteria, Enteric[Title/Abstract])) OR ("Gastrointestinal Microbiome"[Mesh]) | 69,061 |
| #3 | "Liver Cirrhosis"[Mesh] | 97,469 |
| #4 | (((((((Liver Cirrhosis[Title/Abstract]) OR (Hepatic Cirrhosis[Title/Abstract])) OR (Cirrhosis, Hepatic[Title/Abstract])) OR (Cirrhosis, Liver[Title/Abstract])) OR (Fibrosis, Liver[Title/Abstract])) OR (Liver Fibrosis[Title/Abstract])) OR (Cirrhosis[Title/Abstract])) OR ("Liver Cirrhosis"[Mesh]) | 147,474 |
| #5 | "Machine Learning"[Mesh] | 43,589 |
| #6 | ((((((((((((((((((((((((Machine Learning[Title/Abstract]) OR (Learning, Machine[Title/Abstract])) OR (Transfer Learning[Title/Abstract])) OR (Learning, Transfer[Title/Abstract])) OR (Prediction[Title/Abstract])) OR (Random forest[Title/Abstract])) OR (artificial neural network[Title/Abstract])) OR (ANN[Title/Abstract])) OR (Support vector machine[Title/Abstract])) OR (SVM[Title/Abstract])) OR (Gradient Boosting Machine[Title/Abstract])) OR (GBM[Title/Abstract])) OR (XGboost[Title/Abstract])) OR (Decision tree[Title/Abstract])) OR (External validation[Title/Abstract])) OR (classifiers[Title/Abstract])) OR (classifier[Title/Abstract])) OR (logistic regression[Title/Abstract])) OR (Nomogram[Title/Abstract])) OR (Sensitivity[Title/Abstract])) OR (specificity[Title/Abstract])) OR (Confusion matrix[Title/Abstract])) OR (area under the curve[Title/Abstract])) OR (AUC[Title/Abstract])) OR ("Machine Learning"[Mesh]) | 1,936,123 |
| #7 | #2 AND #4 AND #6 | 78 |

**2.Cochrane**

| Search number | Query | Results |
| --- | --- | --- |
| #1 | MeSH descriptor: [Gastrointestinal Microbiome] explode all trees | 893 |
| #2 | (Gastrointestinal Microbiome):ti,ab,kw OR (Gastrointestinal Microbiomes):ti,ab,kw OR (Microbiome, Gastrointestinal):ti,ab,kw OR (Gut Microbiome):ti,ab,kw OR (Gut Microbiomes):ti,ab,kw | 2,394 |
| #3 | (Microbiome, Gut):ti,ab,kw OR (Gut Microflora):ti,ab,kw OR (Microflora, Gut):ti,ab,kw OR (Gut Microbiota):ti,ab,kw OR (Gut Microbiotas):ti,ab,kw | 4,214 |
| #4 | (Microbiota, Gut):ti,ab,kw OR (Gastrointestinal Flora):ti,ab,kw OR (Flora, Gastrointestinal):ti,ab,kw OR (Gut Flora):ti,ab,kw OR (Flora, Gut):ti,ab,kw | 4,008 |
| #5 | (Gastrointestinal Microbiota):ti,ab,kw OR (Gastrointestinal Microbiotas):ti,ab,kw OR (Microbiota, Gastrointestinal):ti,ab,kw OR (Gastrointestinal Microbial Community):ti,ab,kw OR (Gastrointestinal Microbial Communities):ti,ab,kw | 1,779 |
| #6 | (Microbial Community, Gastrointestinal):ti,ab,kw OR (Gastrointestinal Microflora):ti,ab,kw OR (Microflora, Gastrointestinal):ti,ab,kw OR (Gastric Microbiome):ti,ab,kw OR (Gastric Microbiomes):ti,ab,kw | 707 |
| #7 | (Microbiome, Gastric):ti,ab,kw OR (Intestinal Microbiome):ti,ab,kw OR (Intestinal Microbiomes):ti,ab,kw OR (Microbiome, Intestinal):ti,ab,kw OR (Intestinal Microbiota):ti,ab,kw | 2,239 |
| #8 | (Intestinal Microbiotas):ti,ab,kw OR (Microbiota, Intestinal):ti,ab,kw OR (Intestinal Microflora):ti,ab,kw OR (Microflora, Intestinal):ti,ab,kw OR (Intestinal Flora):ti,ab,kw | 3,036 |
| #9 | (Flora, Intestinal):ti,ab,kw OR (Enteric Bacteria):ti,ab,kw OR (Bacteria, Enteric):ti,ab,kw | 1,833 |
| #10 | #1 OR #2 OR #3 OR #4 OR #5 OR #6 OR #7 OR #8 OR #9 | 7,187 |
| #11 | MeSH descriptor: [Liver Cirrhosis] explode all trees | 3,138 |
| #12 | (Liver Cirrhosis):ti,ab,kw OR (Hepatic Cirrhosis):ti,ab,kw OR (Cirrhosis, Hepatic):ti,ab,kw OR (Cirrhosis, Liver):ti,ab,kw OR (Fibrosis, Liver):ti,ab,kw | 10,943 |
| #13 | (Liver Fibrosis):ti,ab,kw OR (Cirrhosis):ti,ab,kw | 12,139 |
| #14 | #11 OR #12 OR #13 | 12,139 |
| #15 | MeSH descriptor: [Machine Learning] explode all trees | 206 |
| #16 | (Machine Learning):ti,ab,kw OR (Learning, Machine):ti,ab,kw OR (Transfer Learning):ti,ab,kw OR (Learning, Transfer):ti,ab,kw OR (Prediction):ti,ab,kw | 16,956 |
| #17 | (Random forest):ti,ab,kw OR (artificial neural network):ti,ab,kw OR (ANN):ti,ab,kw OR (Support vector machine):ti,ab,kw OR (SVM):ti,ab,kw | 2,994 |
| #18 | (Gradient Boosting Machine):ti,ab,kw OR (GBM):ti,ab,kw OR (XGboost):ti,ab,kw OR (Decision tree):ti,ab,kw OR (External validation):ti,ab,kw | 2,781 |
| #19 | (classifiers):ti,ab,kw OR (classifier):ti,ab,kw OR (logistic regression):ti,ab,kw OR (Nomogram):ti,ab,kw OR (Sensitivity):ti,ab,kw | 84,651 |
| #20 | (specificity):ti,ab,kw OR (Confusion matrix):ti,ab,kw OR (area under the curve):ti,ab,kw OR (AUC):ti,ab,kw | 56,984 |
| #21 | #15 OR #16 OR #17 OR #18 OR #19 OR #20 | 136,120 |
| #22 | #10 AND #14 AND #21 | 13 |

**3.Embase**

| Search number | Query | Results |
| --- | --- | --- |
| #1 | 'intestine flora'/exp | 78,187 |
| #2 | 'intestine flora':ti,ab,kw OR 'alimentary canal flora':ti,ab,kw OR 'alimentary tract flora':ti,ab,kw OR 'bowel flora':ti,ab,kw OR 'bowel microbiota':ti,ab,kw OR 'digestive canal flora':ti,ab,kw OR 'digestive tract flora':ti,ab,kw OR 'enteric flora':ti,ab,kw OR 'enteric microbiota':ti,ab,kw OR 'flora, intestine':ti,ab,kw OR 'gastro intestinal flora':ti,ab,kw OR 'gastrointestinal canal flora':ti,ab,kw OR 'gastrointestinal flora':ti,ab,kw OR 'gastrointestinal microbiome':ti,ab,kw OR 'gastrointestinal microbiota':ti,ab,kw OR 'gastrointestinal tract flora':ti,ab,kw OR 'gastrointestine flora':ti,ab,kw OR 'gastrointestine tract flora':ti,ab,kw OR 'gut bacteria':ti,ab,kw OR 'gut microbiota':ti,ab,kw OR 'intestinal bacteria':ti,ab,kw OR 'intestinal bacterial flora':ti,ab,kw OR 'intestinal bacterium':ti,ab,kw OR 'intestinal canal flora':ti,ab,kw OR 'intestinal flora':ti,ab,kw OR 'intestinal microbe':ti,ab,kw OR 'intestinal microbes':ti,ab,kw OR 'intestinal microbiota':ti,ab,kw OR 'intestinal microflora':ti,ab,kw OR 'intestinal microorganism':ti,ab,kw OR 'intestinal tract flora':ti,ab,kw OR 'intestine bacteria':ti,ab,kw OR 'intestine bacteria change':ti,ab,kw OR 'intestine bacterial flora':ti,ab,kw OR 'intestine bacterium':ti,ab,kw OR 'intestine microbial flora':ti,ab,kw OR 'intestine microflora':ti,ab,kw | 63,462 |
| #3 | #1 OR #2 | 93,870 |
| #4 | 'liver cirrhosis'/exp | 191,306 |
| #5 | 'liver cirrhosis':ti,ab,kw OR 'hepatic cirrhosis':ti,ab,kw OR 'cirrhosis, hepatic':ti,ab,kw OR 'cirrhosis, liver':ti,ab,kw OR 'fibrosis, liver':ti,ab,kw OR 'liver fibrosis':ti,ab,kw OR cirrhosis:ti,ab,kw | 186,127 |
| #6 | #4 OR #5 | 243,504 |
| #7 | 'machine learning'/exp | 305,540 |
| #8 | 'machine learning':ti,ab,kw OR 'learning, machine':ti,ab,kw OR 'transfer learning':ti,ab,kw OR 'learning, transfer':ti,ab,kw OR prediction:ti,ab,kw OR 'random forest':ti,ab,kw OR 'artificial neural network':ti,ab,kw OR ann:ti,ab,kw OR 'support vector machine':ti,ab,kw OR svm:ti,ab,kw OR 'gradient boosting machine':ti,ab,kw OR gbm:ti,ab,kw OR xgboost:ti,ab,kw OR 'decision tree':ti,ab,kw OR 'external validation':ti,ab,kw OR classifiers:ti,ab,kw OR classifier:ti,ab,kw OR 'logistic regression':ti,ab,kw OR nomogram:ti,ab,kw OR sensitivity:ti,ab,kw OR specificity:ti,ab,kw OR 'confusion matrix':ti,ab,kw OR 'area under the curve':ti,ab,kw OR auc:ti,ab,kw | 2,593,102 |
| #9 | #7 OR #8 | 2,753,862 |
| #10 | #3 AND #6 AND #9 | 151 |

**4.Web of science**

| Search number | Query | Results |
| --- | --- | --- |
| #1 | Gastrointestinal Microbiome (Topic) or Gastrointestinal Microbiomes (Topic) or Microbiome, Gastrointestinal (Topic) or Gut Microbiome (Topic) or Gut Microbiomes (Topic) or Microbiome, Gut (Topic) or Gut Microflora (Topic) or Microflora, Gut (Topic) or Gut Microbiota (Topic) or Gut Microbiotas (Topic) or Microbiota, Gut (Topic) or Gastrointestinal Flora (Topic) or Flora, Gastrointestinal (Topic) or Gut Flora (Topic) or Flora, Gut (Topic) or Gastrointestinal Microbiota (Topic) or Gastrointestinal Microbiotas (Topic) or Microbiota, Gastrointestinal (Topic) or Gastrointestinal Microbial Community (Topic) or Gastrointestinal Microbial Communities (Topic) or Microbial Community, Gastrointestinal (Topic) or Gastrointestinal Microflora (Topic) or Microflora, Gastrointestinal (Topic) or Gastric Microbiome (Topic) or Gastric Microbiomes (Topic) or Microbiome, Gastric (Topic) or Intestinal Microbiome (Topic) or Intestinal Microbiomes (Topic) or Microbiome, Intestinal (Topic) or Intestinal Microbiota (Topic) or Intestinal Microbiotas (Topic) or Microbiota, Intestinal (Topic) or Intestinal Microflora (Topic) or Microflora, Intestinal (Topic) or Intestinal Flora (Topic) or Flora, Intestinal (Topic) or Enteric Bacteria (Topic) or Bacteria, Enteric (Topic) | 102,760 |
| #2 | intestine flora (Topic) or alimentary canal flora (Topic) or alimentary tract flora (Topic) or bowel flora (Topic) or bowel microbiota (Topic) or digestive canal flora (Topic) or digestive tract flora (Topic) or enteric flora (Topic) or enteric microbiota (Topic) or flora, intestine (Topic) or gastro intestinal flora (Topic) or gastrointestinal canal flora (Topic) or gastrointestinal flora (Topic) or gastrointestinal microbiome (Topic) or gastrointestinal microbiota (Topic) or gastrointestinal tract flora (Topic) or gastrointestine flora (Topic) or gastrointestine tract flora (Topic) or gut bacteria (Topic) or gut microbiota (Topic) or intestinal bacteria (Topic) or intestinal bacterial flora (Topic) or intestinal bacterium (Topic) or intestinal canal flora (Topic) or intestinal flora (Topic) or intestinal microbe (Topic) or intestinal microbes (Topic) or intestinal microbiota (Topic) or intestinal microflora (Topic) or intestinal microorganism (Topic) or intestinal tract flora (Topic) or intestine bacteria (Topic) or intestine bacteria change (Topic) or intestine bacterial flora (Topic) or intestine bacterium (Topic) or intestine microbial flora (Topic) or intestine microflora (Topic) | 115,344 |
| #3 | Liver Cirrhosis (Topic) or Hepatic Cirrhosis (Topic) or Cirrhosis, Hepatic (Topic) or Cirrhosis, Liver (Topic) or Fibrosis, Liver (Topic) or Liver Fibrosis (Topic) or Cirrhosis (Topic) | 154,157 |
| #4 | Machine Learning (Topic) or Learning, Machine (Topic) or Transfer Learning (Topic) or Learning, Transfer (Topic) or Prediction (Topic) or Random forest (Topic) or artificial neural network (Topic) or ANN (Topic) or Support vector machine (Topic) or SVM (Topic) or Gradient Boosting Machine (Topic) or GBM (Topic) or XGboost (Topic) or Decision tree (Topic) or External validation (Topic) or classifiers (Topic) or classifier (Topic) or logistic regression (Topic) or Nomogram (Topic) or Sensitivity (Topic) or specificity (Topic) or Confusion matrix (Topic) or area under the curve (Topic) or AUC (Topic) | 4,201,569 |
| #5 | #1 AND #3 AND #4 | 181 |
| #6 | #2 AND #3 AND #4 | 160 |
